# Preventive effect of small‐leaved Kuding tea (*Ligustrum robustum*) on high‐diet‐induced obesity in C57BL/6J mice

**DOI:** 10.1002/fsn3.1758

**Published:** 2020-07-08

**Authors:** Ya Wu, Jun Yang, Xiaojing Liu, Ying Zhang, Ailing Lei, Ruokun Yi, Fang Tan, Xin Zhao

**Affiliations:** ^1^ Chongqing Collaborative Innovation Center for Functional Food Chongqing University of Education Chongqing China; ^2^ Chongqing Engineering Research Center of Functional Food Chongqing University of Education Chongqing China; ^3^ Chongqing Engineering Laboratory for Research and Development of Functional Food Chongqing University of Education Chongqing China; ^4^ College of Biological and Chemical Engineering Chongqing University of Education Chongqing China; ^5^ Department of Gastroenterology People's Hospital of Chongqing Banan District Chongqing China; ^6^ Department of Public Health Our Lady of Fatima University Valenzuela Philippines

**Keywords:** anti‐inflammation, anti‐obesity, high‐fat diet, *Ligustrum robustum*, lipid metabolism, small‐leaved Kuding tea

## Abstract

Small‐leaved Kuding tea (SLKDT; *Ligustrum robustum*) is a traditional Chinese tea. We systematically investigated the effect of SLKDT extract on obesity. SLKDT‐controlled weight gain in mice fed a high‐fat diet. Tissue specimen results showed that the SLKDT extract alleviated liver damage and fat accumulation. Meanwhile, SLKDT extract improved dyslipidemia by decreasing total cholesterol, triglycerides, and low‐density lipoprotein cholesterol levels and increasing high‐density lipoprotein cholesterol levels. Furthermore, SLKDT extract reduced alanine aminotransferase, alkaline phosphatase, and aspartate transaminase levels in the serum and liver tissues; decreased inflammatory cytokines, including interleukin (IL)‐1β, tumor necrosis factor‐α, interferon‐γ, and IL‐6; and increased the anti‐inflammatory cytokines, IL‐4 and IL‐10. The quantitative PCR results showed that SLKDT extract upregulated the mRNA expressions of peroxisome proliferator‐activated receptor (PPAR)‐α, lipoprotein lipase, carnitine palmitoyltransferase 1, and cholesterol 7 alpha hydroxylase and downregulated PPAR‐γ and CCAAT/enhancer‐binding protein‐alpha mRNA expressions in the obese mouse livers to reduce adipocyte differentiation and fat accumulation, promote fat oxidation, and improve dyslipidemia, thereby inhibiting the immune response and alleviating liver injury. SLKDT shows a potential for preventing obesity and regulating obesity‐related syndrome, so it is possible to be further developed as a novel treatment for fighting obesity.

## Graphical abstract

Small‐leaved Kuding tea contains neochlorogenic acid, chlorogenic acid, cryptochlorogenic acid, isochlorogenic acid A, isochlorogenic acid B, isochlorogenic acid C, and rutin. These ingredients play a role in weight loss.

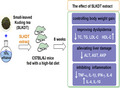

## 1 INTRODUCTION

As living standards are improving, the obese population is increasing drastically. Obesity is mainly manifested as excessive or abnormal fat accumulation (Thomas, Frost, Taylor‐Robinson, & Bell, 2012), and it has become a serious global health problem. Excess energy intake (Vandevijvere, Chow, Hall, Umaliet, & Swinburn, 2015), insulin resistance (Kahn, Hull, & Utzschneider, 2006), inflammatory response (Saltiel & Olefsky, 2017), and oxidative stress (Savini, Gasperi, & Catani, 2016) are all possible mechanisms by which obesity occurs and develops. Many studies have shown that obesity can cause lipid and glucose metabolism disorders, thus inducing metabolic diseases such as diabetes, hypertension, atherosclerosis, and non‐alcoholic fatty liver (Boulange, Neves, Chilloux, Nicholson, & Dumas, 2016; Despres & Lemieux, 2006; Haslam & James, 2005; Mozaffarian, 2016). Obesity seriously threatens peoples’ health and living quality and increases financial burdens on families. Therefore, it is extremely important to study how to effectively prevent and control the occurrence of obesity and reduce the risk of its associated metabolic diseases.

Kuding tea is a traditional drink with a history spanning more than 2,000 years in China. It is mainly distributed in southwestern China (Sichuan, Chongqing, Guizhou, Hunan, and Hubei) and southern China (Jiangxi, Yunnan, Guangdong, Fujian, and Hainan) (Zhu et al., 2009). Kuding teas include large‐leaved Kuding tea (*Ilex latifolia* Thunb and *Ilex kudingcha* C.J. Tseng) and small‐leaved Kuding tea (SLKDT; *Ligustrum robustum*) (Li, Xu, et al., 2013). Kuding tea tastes fragrant and bitter, then sweet and cool. It is a natural drink with medicinal properties. Kuding tea is rich in natural active components such as triterpenoids (Wang et al., 2012), polyphenols, and flavonoids (Liu et al., 2009); thus, it has remarkable biological activity and can regulate lipid metabolism (Fan et al., 2012), protect the cardiovascular and cerebrovascular systems (Chen, Li, & Xie, 1995), and exert hypoglycemic (Song, Xie, Zhou, Yu, & Fang, 2012), antioxidant (Zhang, Xu, Sun, Ye, & Zeng, 2010), and antibacterial effects (Lu & Huang, 2009). SLKDT is a common drink in daily life and is easily accepted by consumers. Compared with most tea leaves, SLKDT has high selenium and low caffeine contents (Yang & Zhu, 1996) and possesses good medicinal activity and health value. Studies have revealed the effects of SLKDT on hypolipidemia and weight loss (Xie et al., 2015; Yang et al., 2015), but systematic research on the anti‐obesity effects and mechanisms of SLKDT remains limited. Therefore, it is extremely significant to deeply study the active components of SLKDT and its anti‐obesity effects and mechanisms, to develop SLKDT as a functional drug or food for preventing and treating obesity.

In this study, obesity models were established by a high‐fat diets, and SLKDT extract was used to intervene. We observed body weights and pathological sections and measured the relevant biochemical indicators in the serum and tissue to verify the effect of SLKDT. Lipid metabolism‐related gene expressions were tested to clarify the SLKDT mechanism of action. This investigation provided a theoretical basis and experimental support for developing SLKDT as a natural and efficient product for preventing and treating obesity.

## 2 MATERIALS AND METHODS

### 2.1 Preparation of the SLKDT extract

Dried SLKDT (100 g, from Hainan Province) was weighed and pulverized, and then 70% ethanol was added at a liquid/material ratio of 20:1. The mixed liquid was heated for 3 hr at 60°C, then cooled and filtered to obtain the crude extract. The extraction solution was purified via FL‐3 macroporous resin with 70% ethanol as the eluant (Mao, Liu, & Ran, 2011). When the eluant became colorless, the eluted solution was collected and evaporated. The residue was dried and smashed to obtain the SLKDT extract.

### 2.2 Analysis of the SLKDT extract composition

Neochlorogenic acid (1.2 mg), rutin (1.5 mg), chlorogenic acid (1.2 mg), quercetin (1.7 mg), kaempferitrin (1.8 mg), isochlorogenic acid B (1.3 mg), cryptochlorogenic acid (1.2 mg), isochlorogenic acid A (1.3 mg), rhamnetin (0.7 mg), and isochlorogenic acid C (2.2 mg) (Beijing Putian Tongchuang Biological Technology Co., Ltd.) were separately weighed, and methanol (HPLC grade) was added to prepare 1 mg/ml standard stock solutions. Standard stock solutions were mixed together, and then a mixed standard solution was obtained. SLKDT extract (10 mg) was dissolved in 1 ml of dimethyl sulfoxide, and then 1.5 ml of methanol and 1.5 ml of H_2_O were added. Samples were filtered through a microporous membrane (0.22 μm) to afford the test solution.

Separation chromatography was performed using an UltiMate3000 HPLC System (Thermo Fisher Scientific) and a Thermo Scientific Accucore C18 column (150 × 1.6 mm, 2.6 µm). Mobile phase A was acetonitrile (HPLC grade); mobile phase B was 0.5% glacial acetic acid aqueous solution. The mobile phase gradient was 0 min, 12% A; 0–15 min, 20% A; 15–35 min, 40% A; 35–45 min, 70% A; and 45–50 min, 100% A. The flow rate was 0.6 ml/min; the column temperature was 30°C; the detection wavelength was 359 nm; and the injection volume was 10 μl. Polyphenols were determined according to retention time and analyzed via the each component's chromatographic peak area.

### 2.3 Animal models and treatment

Fifty healthy C57BL/6J mice (weighing 20 ± 2 g, half male and female) were acclimated for 1 week, then randomly divided into the normal, model, L‐carnitine, low‐concentration SLKDT extract (SLKDT‐L), and high‐concentration SLKDT extract (SLKDT‐H) groups (*n* = 10/group [5 males and 5 females] per group). The normal group was supplied with drinking water and normal maintenance food; the other groups were fed a D1249251 high‐fat diet (Chongqing Medical University). Mice in the L‐carnitine group were intragastrically administered 200 mg/kg L‐carnitine daily; mice in the SLKDT‐L and SLKDT‐H groups were administered, respectively, 100 mg/kg and 200 mg/kg SLKDT extract by gavage daily. After 4 weeks, except the normal group, other groups were switched from regular drinking water to 10% sugar water. Eight weeks later, all mice were fasted for 24 hr and then euthanized. Blood was obtained via the orbits, and the livers and epididymal fat (from the males) were collected and recorded for further experiments. The formula was used to calculate the organ index: Organ index = Organ weight (g)/Mouse body weight (g) × 100.

### 2.4 Histological analysis of the liver and epididymal fat

Pieces of both the liver and epididymal fat (~0.5 cm^2^ each) were fixed in 10% formalin solution for 48 hr, then dehydrated, routinely processed, embedded in paraffin, sectioned, and stained with hematoxylin and eosin. Tissue histology was examined via an optical microscope (BX43 microscope; Olympus).

### 2.5 Measurements of TC, TG, HDL‐C, and LDL‐C levels in the serum and liver

Serum was obtained by centrifuging the plasma at 730 *g* for 10 min, and then the supernatant was collected. Serum total cholesterol (TC), triglycerides (TG), low‐density lipoprotein cholesterol (LDL‐C), and high‐density lipoprotein cholesterol (HDL‐C) levels were measured via kits following the manufacturer's instructions (Nanjing Jiancheng Bioengineering Institute).

Liver homogenate (10%) was centrifuged at 4,000 rpm for 10 min to obtain the supernatant. TC, TG, LDL‐C, and HDL‐C levels in liver were determined following each kit's instructions (Nanjing Jiancheng Bioengineering Institute).

### 2.6 Measurements of AKP, ALT, and AST levels in the serum and liver

Serum was obtained by centrifuging the plasma at 4,000 rpm for 10 min, and then the upper light‐yellow liquid was collected. Alanine aminotransferase (ALT), aspartate transaminase (AST), and alkaline phosphatase (AKP) levels in serum were analyzed using kits (Nanjing Jiancheng Bioengineering Institute).

Liver homogenate (10%) was centrifuged at 4,000 rpm for 10 min to afford the supernatant. The levels of ALT, AST, and AKP in the liver were analyzed according to each kit's instructions (Nanjing Jiancheng Bioengineering Institute).

### 2.7 Determination of serum cytokine TNF‐α, IFN‐γ, IL‐6, IL‐1β, IL‐4, and IL‐10 levels

Serum was obtained by centrifuging the plasma at 4,000 rpm for 10 min to collect the supernatant. The cytokine levels of interferon gamma (IFN‐γ), tumor necrosis factor‐alpha (TNF‐α), interleukin (IL)‐4, IL‐1β, IL‐6, and IL‐10 in serum were analyzed following each kit's instructions (Beijing Chenglin Biotechnology Co., Ltd.).

### 2.8 Quantitative PCR assay

Liver tissue was homogenized, and 1 ml of RNAzol reagent (Invitrogen) was used to extract the total RNA from the liver. And then the total RNA extracted was diluted to the concentration of 1 μg/μl. One microliter of diluted total RNA extract was utilized for reverse transcription to synthesize the cDNA template as per the kit instructions (Tiangen Biotech Co., Ltd.). Ten microliter of SYBR Green PCR Master Mix and 1 μl of the upstream and downstream primers were added to the cDNA template (Table 1). The quantitative PCR cycles were performed under the condition: 95°C for 30 s, then 40 cycles of 95°C for 30 s, 55°C for 30 s, 72°C for 30 s, and a final extension at 95°C for 30 s, followed by 55°C for 35 s. The 2^−ΔΔ^ *^Ct^* method was utilized to calculate the relative gene expressions, in which GADPH served as the internal reference.

**TABLE 1:** Sequences of primers used in this study

Gene name	Sequence
*CYP7A1*	Forward: 5′‐AGCAACTAAACAACCTGCCAGTACTA‐3′ Reverse: 5′‐GTCCGGATATTCAAGGATGCA‐3′
*PPAR‐γ*	Forward: 5′‐AGGCCGAGAAGGAGAAGCTGTTG‐3′ Reverse:5′‐TGGCCACCTCTTTGCTGTGCTC‐3′
*PPAR‐α*	Forward: 5′‐CCTCAGGGTACCACTACGGAGT‐3′ Reverse: 5′‐GCCGAATAGTTCGCCGAA‐3′
*CPT1*	Forward: 5′‐AAAGATCAATCGGACCCTAGACA‐3′ Reverse: 5′‐CAGCGAGTAGCGCATAGTCA‐3′
*LPL*	Forward: 5′‐AGGGCTCTGCCTGAGTTGTA‐3′ Reverse: 5′‐CAGCGAGTAGCGCATAGTCA‐3′
*C/EBP‐α*	Forward: 5′‐TGGACAAGAACAGCAACGAGTAC‐3′ Reverse: 5′‐GCAGTTGCCCATGGCCTTGAC‐3′
*GAPDH*	Forward: 5′‐ACCCAGAAGACTGTGGATGG‐3′ Reverse: 5′‐ACACATTGGGGGTAGGAACA‐3′

Abbreviations: C/EBP‐α, CCAAT/enhances binding protein alpha; CPT1, carnitine palmitoyltransferase 1; CYP7A1, cholesterol 7 alpha hydroxylase; GADPH, glyceraldehyde‐3‐phosphate dehydrogenase; LPL, lipoprotein lipase; PPAR‐α, peroxisome proliferator‐activated receptor alpha; PPAR‐γ, peroxisome proliferator‐activated receptor gamma.

### 2.9 Statistical analysis

The SPSS 17.0 (SPSS) and GraphPad Prism 7 statistical software (GraphPad Software Inc.) were used to analyze the data. Experimental results are expressed as the mean ± *SD*. One‐way analysis of variance or a *t* test were used for between‐group comparisons. *p* < .05 was considered statistically significant.

## 3 RESULTS

### 3.1 Compositional analysis of the SLKDT extract

**FIGURE 1:**
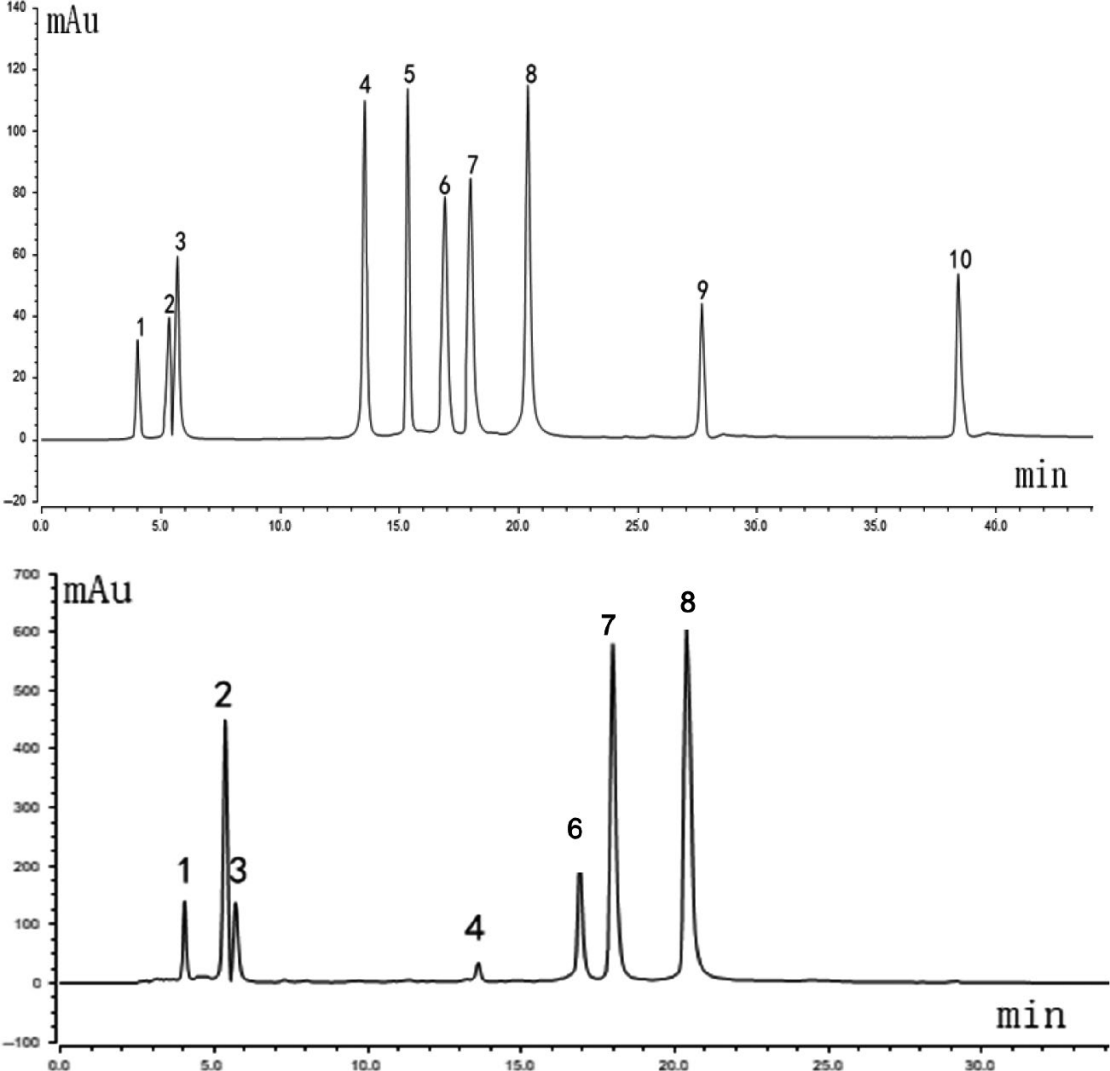
Polyphenols constituents of SLKDT extract. Standard chromatograms (up); SLKDT extract polyphenols chromatograms (down): 1. neochlorogenic acid; 2. chlorogenic acid; 3. cryptochlorogenic acid; 4. rutin; 5. kaempferitrin; 6. isochlorogenic acid B; 7. isochlorogenic acid A; 8. isochlorogenic acid C; 9. quercetin; 10. rhamnetin. SLKDT, small‐leaved Kuding tea

The HPLC results showed that the chlorogenic acid and its derivatives were the main polyphenols in SLKDT extract (Figure 1). The standard spectral data showed that the polyphenol contents were neochlorogenic acid (11.199 mg/g), chlorogenic acid (10.837 mg/g), cryptochlorogenic acid (8.874 mg/g), isochlorogenic acid A (11.124 mg/g), isochlorogenic acid C (11.840 mg/g), isochlorogenic acid B (14.967 mg/g), and rutin (0.454 mg/g). Quercetin, rhamnetin and kaempferitrin were undetected.

### 3.2 Effect of the SLKDT extract on mouse body weight

**FIGURE 2:**
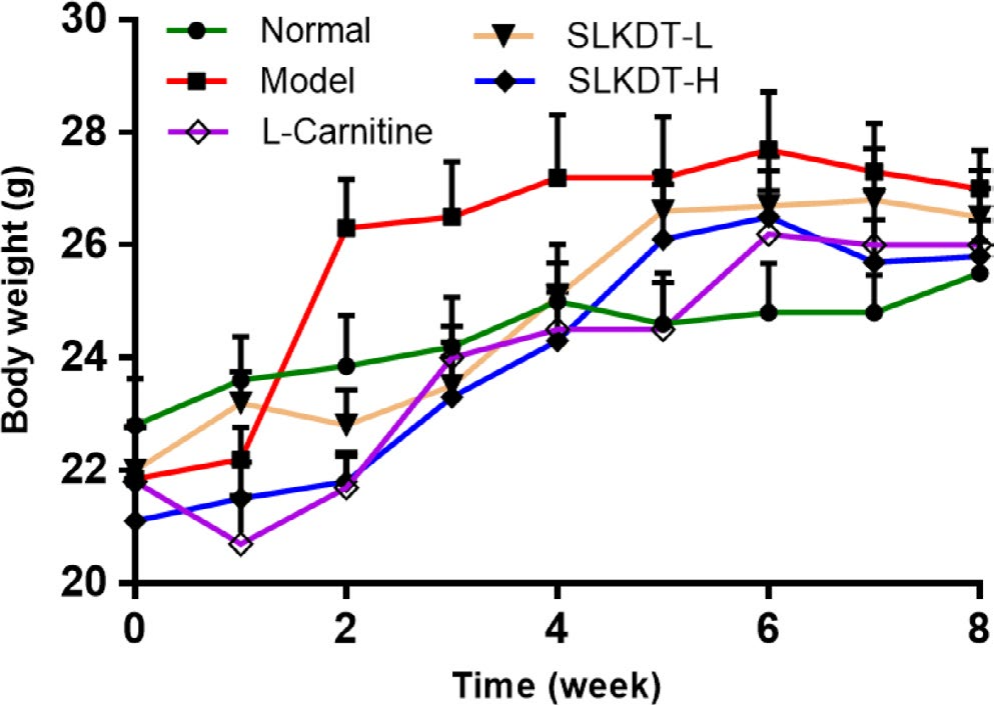
Body weight gain. The data are shown as mean ± *SD* (*n* = 10). L‐carnitine, mice treated with 200 mg/kg L‐carnitine; SLKDT‐L, mice treated with 100 mg/kg SLKDT extract; SLKDT‐H, mice treated with 200 mg/kg SLKDT extract. SLKDT, small‐leaved Kuding tea

Weight gain increased dramatically in the model group in the early stages but increased gradually in the normal group throughout the test period (Figure 2). After experiment, the body weight of model group was obviously higher than that of normal group (*p* < .05). And compared with the model group, the body weight of mice in L‐carnitine group and SLKD‐H was decreased (*p* < .05). The effect of SLKDT‐H was similar to that of L‐carnitine.

### 3.3 Liver and epididymal fat indexes

The liver and epididymal fat indexes are shown in Table 2. The data vary between different groups, but there is no significant difference between the groups. It may be that the weight of the organs did not change significantly in this experiment, so the treatment group did not show obvious effects.

**TABLE 2:** Liver and epididymal fat indexes of mice in each group

Group	Normal	Model	L‐carnitine	SLKDT‐L	SLKDT‐H
Liver index	4.07 ± 0.27^a^, ^a–d^	4.20 ± 0.23^a^, ^a–d^	4.12 ± 0.17^a^, ^a–d^	4.18 ± 0.25^a^, ^a–d^	4.15 ± 0.17^a^, ^a–d^
Epididymal fat index	1.58 ± 0.49^a^, ^a–d^	2.22 ± 0.75^a^, ^a–d^	1.89 ± 0.57^a^, ^a–d^	1.97 ± 0.40^a^, ^a–d^	1.62 ± 0.71^a^, ^a–d^

Note

Values presented are the mean ± *SD* (liver index: *n* = 10/group, epididymal fat index: *n* = 5/group). L‐carnitine, mice treated with 200 mg/kg L‐carnitine; SLKDT‐L, mice treated with 100 mg/kg SLKDT extract; SLKDT‐H, mice treated with 200 mg/kg SLKDT extract.

Abbreviation: SLKDT, small‐leaved Kuding tea.

^a^ Mean values with different superscript letters are significantly different (*p* < .05).

### 3.4 Histopathological examination of the liver and epididymal fat

**FIGURE 3:**
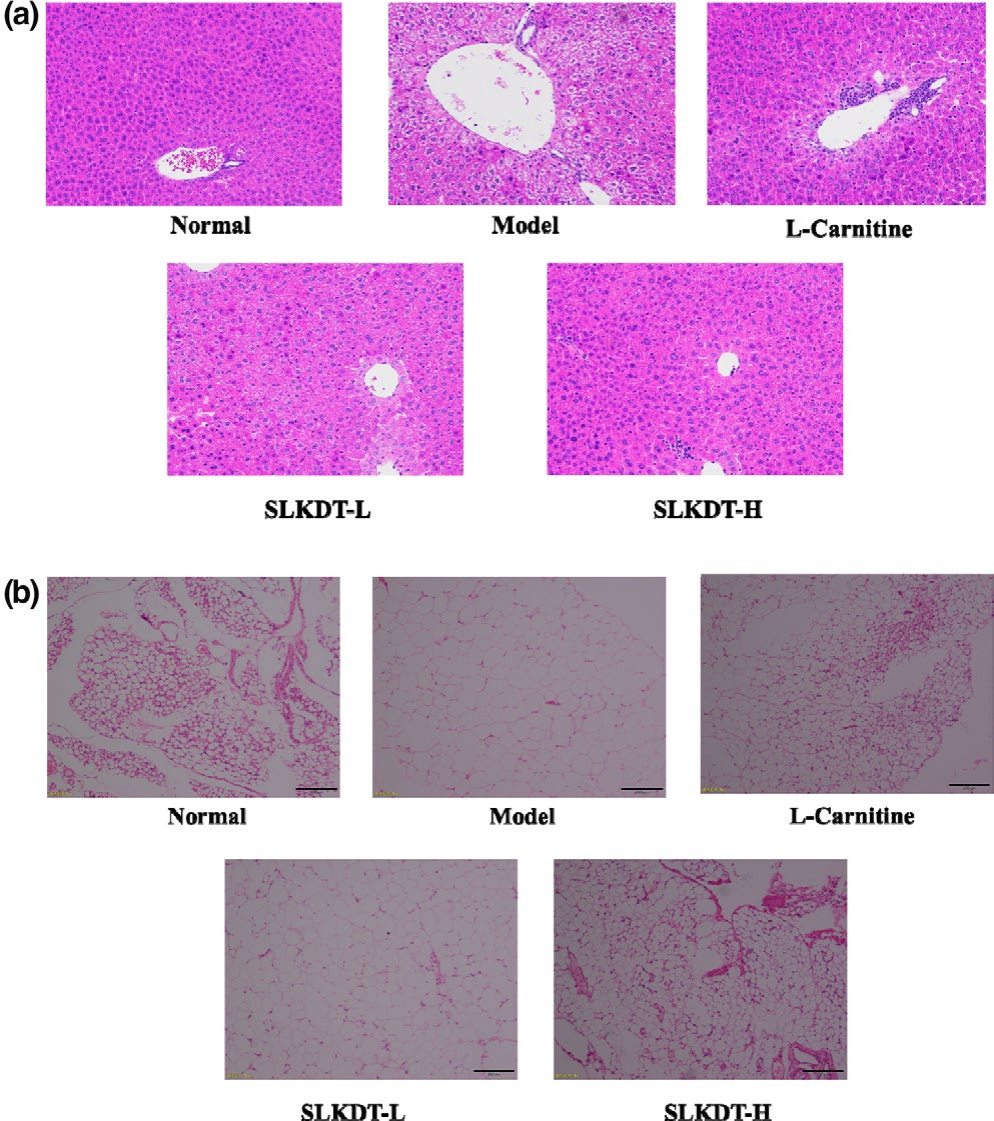
(a) Histopathological observation of liver sections in mice of the different groups after staining with hematoxylin and eosin (H&E). (b) H&E histopathological observation of the epididymal fat tissue in mice of the different groups. SLKDT, small‐leaved Kuding tea

Figure 3a shows that the hepatocyte boundaries in the normal group were clear, with a few small vacuoles. Compared with the normal group, hepatocytes in the model group were large and swollen, and the number of lipid droplets was significantly increased. After treatment with L‐carnitine and SLKDT, the number of lipid droplets in hepatocyte was reduced, and hepatocyte was arranged in an orderly arrangement, which has cellular morphology. The SLKDT‐H extract was more effective than was the SLKDT‐L extract.

Figure 3b shows that the adipocytes of the epididymal fat sections were large and highly saturated compared with those of the normal group. After treatment, the adipocytes in the L‐carnitine and SLKDT groups were smaller than those of the model group. The shape of the adipocytes in the SLKDT‐H group was nearly the same as that in the normal group, revealing that the high concentration has better performance.

### 3.5 TC, TG, LDL‐C, and HDL‐C levels in the mouse serum and liver tissue

Blood lipid levels can reflect systemic lipid metabolism. The levels of TC, TG, and LDL‐C in serum and liver tissue of mice in the model group were increased, and the HDL‐C level was decreased compared with those of the normal group (Tables 3 and 4; *p* < .05), thus revealing an abnormal lipid metabolism. The SLKDT extract significantly increased the HDL‐C level and decreased the TC, TG, and LDL‐C levels in the serum and liver tissues (*p* < .05). The effect of SLKDT‐H was comparable with that of L‐carnitine as a whole.

**TABLE 3:** Serum levels of TG, TC, HDL‐C, and LDL‐C in mice of each groups

Group	TC (mmol/L)	TG (mmol/L)	HDL‐C (mmol/L)	LDL‐C (mmol/L)
Normal	5.48 ± 0.34^a^, ^a–d^	4.54 ± 0.24^a^, ^a–d^	2.78 ± 0.15^a^, ^a–d^	1.25 ± 0.06^a^, ^a–d^
Model	6.75 ± 0.33^a^, ^a–d^	5.97 ± 0.70^a^, ^a–d^	1.94 ± 0.26^a^, ^a–d^	2.07 ± 0.35^a^, ^a–d^
L‐carnitine	6.01 ± 0.30^a^, ^a–d^	4.82 ± 0.28^a^, ^a–d^	2.25 ± 0.27^a^, ^a–d^	1.54 ± 0.04^a^, ^a–d^
SLKDT‐L	5.88 ± 0.27^a^, ^a–d^	5.47 ± 0.43^a^, ^a–d^	2.29 ± 0.07^a^, ^a–d^	1.60 ± 0.26^a^, ^a–d^
SLKDT‐H	5.72 ± 0.17^a^, ^a–d^	5.35 ± 0.16^a^, ^a–d^	2.34 ± 0.14^a^, ^a–d^	1.37 ± 0.16^a^, ^a–d^

Note

Values presented are the mean ± *SD* (*n* = 10/group). L‐carnitine, mice treated with 200 mg/kg L‐carnitine; SLKDT‐L, mice treated with 100 mg/kg SLKDT extract; SLKDT‐H, mice treated with 200 mg/kg SLKDT extract.

Abbreviations: HDL‐C, high‐density lipoprotein cholesterol; LDL‐C, low‐density lipoprotein cholesterol; SLKDT, small‐leaved Kuding tea; TC, total cholesterol; TG, triglycerides.

^a–d^ Mean values with different letters over the same column are significantly different (*p* < .05).

**TABLE 4:** Liver tissue levels of TG, TC, HDL‐C, and LDL‐C in mice of each groups

Group	TC (mmol/gprot)	TG (mmol/gprot)	HDL‐C (mmol/gprot)	LDLC (mmol/gprot)
Normal	22.88 ± 0.58^a–d^, ^a–c^	2.15 ± 0.45^a–d^, ^a–c^	19.20 ± 0.63^a–d^, ^a–c^	3.59 ± 0.15^a–d^, ^a–c^
Model	32.39 ± 5.04^a–d^, ^a–c^	3.42 ± 0.37^a–d^, ^a–c^	13.51 ± 0.90^a–d^, ^a–c^	5.52 ± 0.34^a–d^, ^a–c^
L‐carnitine	29.39 ± 5.66^a–d^, ^a–c^	2.83 ± 0.26^a–d^, ^a–c^	15.47 ± 0.86^a–d^, ^a–c^	4.81 ± 0.21^a–d^, ^a–c^
SLKDT‐L	26.38 ± 3.83^a–d^, ^a–c^	2.65 ± 0.17^a–d^, ^a–c^	16.27 ± 1.91^a–d^, ^a–c^	4.09 ± 0.23^a–d^, ^a–c^
SLKDT‐H	23.87 ± 1.29^a–d^, ^a–c^	2.63 ± 0.21^a–d^, ^a–c^	16.43 ± 1.48^a–d^, ^a–c^	3.73 ± 0.24^a–d^, ^a–c^

Note

Values presented are the mean ± *SD* (*n* = 10/group). L‐carnitine, mice treated with 200 mg/kg L‐carnitine; SLKDT‐L, mice treated with 100 mg/kg SLKDT extract; SLKDT‐H, mice treated with 200 mg/kg SLKDT extract.

Abbreviations: HDL‐C, high‐density lipoprotein cholesterol; LDL‐C, low‐density lipoprotein cholesterol; SLKDT, small‐leaved Kuding tea; TC, total cholesterol; TG, triglycerides.

^a–d^ Mean values with different letters over the same column are significantly different (*p* < .05).

### 3.6 ALT, AST, and AKP levels in the mouse serum and liver tissues

ALT, AST, and ALP levels, as enzymatic indicators of liver function, can reflect the degree of liver damage. The levels of ALT, AST, and AKP in serum and liver were highest in the model group, indicating liver damage in the obese mice (Tables 5 and 6). Treatment with SLKDT and L‐carnitine significantly decreased the levels of ALT, AST, and AKP (*p* < .05). The effect of SLKDT‐H was similar to the levels of the normal group.

**TABLE 5:** Serum levels of AST, ALT, and AKP in mice of each groups

Group	AST (U/L)	ALT (U/L)	AKP (U/L)
Normal	42.75 ± 4.39^a–d^	10.09 ± 3.28^a–d^	5.90 ± 0.20^a–d^
Model	72.61 ± 9.52^a–d^	25.27 ± 7.51^a–d^	11.16 ± 0.97^a–d^
L‐carnitine	54.51 ± 10.20^a–d^	10.78 ± 1.38^a–d^	6.95 ± 0.73^a–d^
SLKDT‐L	53.28 ± 4.84^a–d^	14.74 ± 3.11^a–d^	7.30 ± 0.90^a–d^
SLKDT‐H	51.54 ± 1.76^a–d^	10.81 ± 2.15^a–d^	6.75 ± 0.99^a–d^

Note

Values presented are the mean ± *SD* (*n* = 10/group). L‐carnitine, mice treated with 200 mg/kg L‐carnitine; SLKDT‐L, mice treated with 100 mg/kg SLKDT extract; SLKDT‐H, mice treated with 200 mg/kg SLKDT extract.

Abbreviations: AKP, alkaline phosphatase; ALT, alanine aminotransferase; AST, aspartate transaminase; SLKDT, small‐leaved Kuding tea.

^a–d^ Mean values with different letters over the same column are significantly different (*p* < .05).

**TABLE 6:** Liver tissue levels of AST, ALT, and AKP in mice of each groups

Group	AST (U/gprot)	ALT (U/gprot)	AKP (U/gprot)
Normal	45.85 ± 1.60^a–d^	185.32 ± 12.92^a–d^	82.79 ± 4.22^a–d^
Model	80.63 ± 5.36^a–d^	289.60 ± 13.69^a–d^	162.58 ± 8.56^a–d^
L‐carnitine	64.51 ± 9.79^a–d^	198.56 ± 9.10^a–d^	96.49 ± 9.13^a–d^
SLKDT‐L	58.51 ± 13.50^a–d^	202.81 ± 2.87^a–d^	134.88 ± 8.65^a–d^
SLKDT‐H	55.95 ± 6.27^a–d^	199.17 ± 10.27^a–d^	89.22 ± 5.51^a–d^

Note

Values presented are the mean ± *SD* (*n* = 10/group). L‐carnitine, mice treated with 200 mg/kg L‐carnitine; SLKDT‐L, mice treated with 100 mg/kg SLKDT extract; SLKDT‐H, mice treated with 200 mg/kg SLKDT extract.

Abbreviations: AKP, alkaline phosphatase; ALT, alanine aminotransferase; AST, aspartate transaminase; SLKDT, small‐leaved Kuding tea.

^a–d^ Mean values with different letters over the same column are significantly different (*p* < .05).

### 3.7 The serum cytokine TNF‐α, IL‐1β, IFN‐γ, IL‐6, IL‐4, and IL‐10 levels in mice

Obesity is a state of endocrine dysfunction among adipocytes, which can induce metabolic inflammation in adipose tissue and gradually evolve into chronic low‐level inflammation. Compared with those of the normal group, the levels of proinflammatory factors, TNF‐α, IL‐6, IFN‐γ, and IL‐1β increased significantly, while the levels of anti‐inflammatory factors IL‐4 and IL‐10 decreased in the model group (Tables 7 and 8; *p* < .05), indicating that the mice were in an inflammatory state. SLKDT and L‐carnitine intervention significantly decreased the IL‐6, TNF‐α, IFN‐γ, and IL‐1β levels and increased the IL‐4 and IL‐10 levels (*p* < .05), indicating that SLKDT and L‐carnitine inhibited the immune response. On the whole, the cytokine levels in SLKDT‐H group were close to those in the normal group, and slightly better than those in L‐carnitine group.

**TABLE 7:** Serum levels of the cytokines TNF‐α, IFN‐γ, IL‐1β, and IL‐6 in mice of each groups

Group	IL‐6 (pg/mL)	IL‐1β (pg/mL)	TNF‐α (pg/mL)	IFN‐γ (pg/mL)
Normal	38.78 ± 1.09^a–d^	29.21 ± 0.36^a–d^	172.46 ± 7.73^a–d^	243.86 ± 3.89^a–d^
Model	50.68 ± 1.76^a–d^	32.87 ± 0.68^a–d^	257.56 ± 12.34^a–d^	315.05 ± 2.02^a–d^
L‐carnitine	47.78 ± 1.20^a–d^	30.29 ± 0.72^a–d^	221.84 ± 8.03^a–d^	267.31 ± 20.94^a–d^
SLKDT‐L	44.38 ± 1.05^a–d^	30.79 ± 0.23^a–d^	223.39 ± 9.12^a–d^	295.52 ± 9.43^a–d^
SLKDT‐H	43.05 ± 1.47^a–d^	30.27 ± 2.16^a–d^	177.65 ± 7.69^a–d^	248.74 ± 12.85^a–d^

Note

Values presented are the mean ± *SD* (*n* = 10/group). L‐carnitine, mice treated with 200 mg/kg L‐carnitine; SLKDT‐L, mice treated with 100 mg/kg SLKDT extract; SLKDT‐H, mice treated with 200 mg/kg SLKDT extract.

Abbreviations: IFN‐γ, interferon gamma; IL, interleukin; SLKDT, small‐leaved Kuding tea; TNF‐α, tumor necrosis factor‐alpha.

^a–d^ Mean values with different letters over the same column are significantly different (*p* < .05).

**TABLE 8:** Serum levels of the cytokines IL‐10 and IL‐4 in mice of each groups

Group	IL‐4 (pg/ml)	IL‐10 (pg/ml)
Normal	70.31 ± 1.13^a–d^, ^a–c^	247.24 ± 16.95^a–d^, ^a–c^
Model	53.72 ± 0.92^a–d^, ^a–c^	183.34 ± 13.78^a–d^, ^a–c^
L‐carnitine	66.76 ± 0.26^a–d^, ^a–c^	215.86 ± 15.19^a–d^, ^a–c^
SLKDT‐L	65.66 ± 1.37^a–d^, ^a–c^	202.65 ± 13.09^a–d^, ^a–c^
SLKDT‐H	66.06 ± 1.20^a–d^, ^a–c^	217.40 ± 11.46^a–d^, ^a–c^

Note

Values presented are the mean ± *SD* (*n* = 10/group). L‐carnitine, mice treated with 200 mg/kg L‐carnitine; SLKDT‐L, mice treated with 100 mg/kg SLKDT extract; SLKDT‐H, mice treated with 200 mg/kg SLKDT extract.

Abbreviation: IL, interleukin; SLKDT, small‐leaved Kuding tea.

^a–c^ Mean values with different letters over the same column are significantly different (*p* < .05).

### 3.8 PPAR‐γ, C/EBP‐α, PPAR‐α, LPL, CPT1, and CYP7A1 mRNA expressions in the liver tissue

**FIGURE 4:**
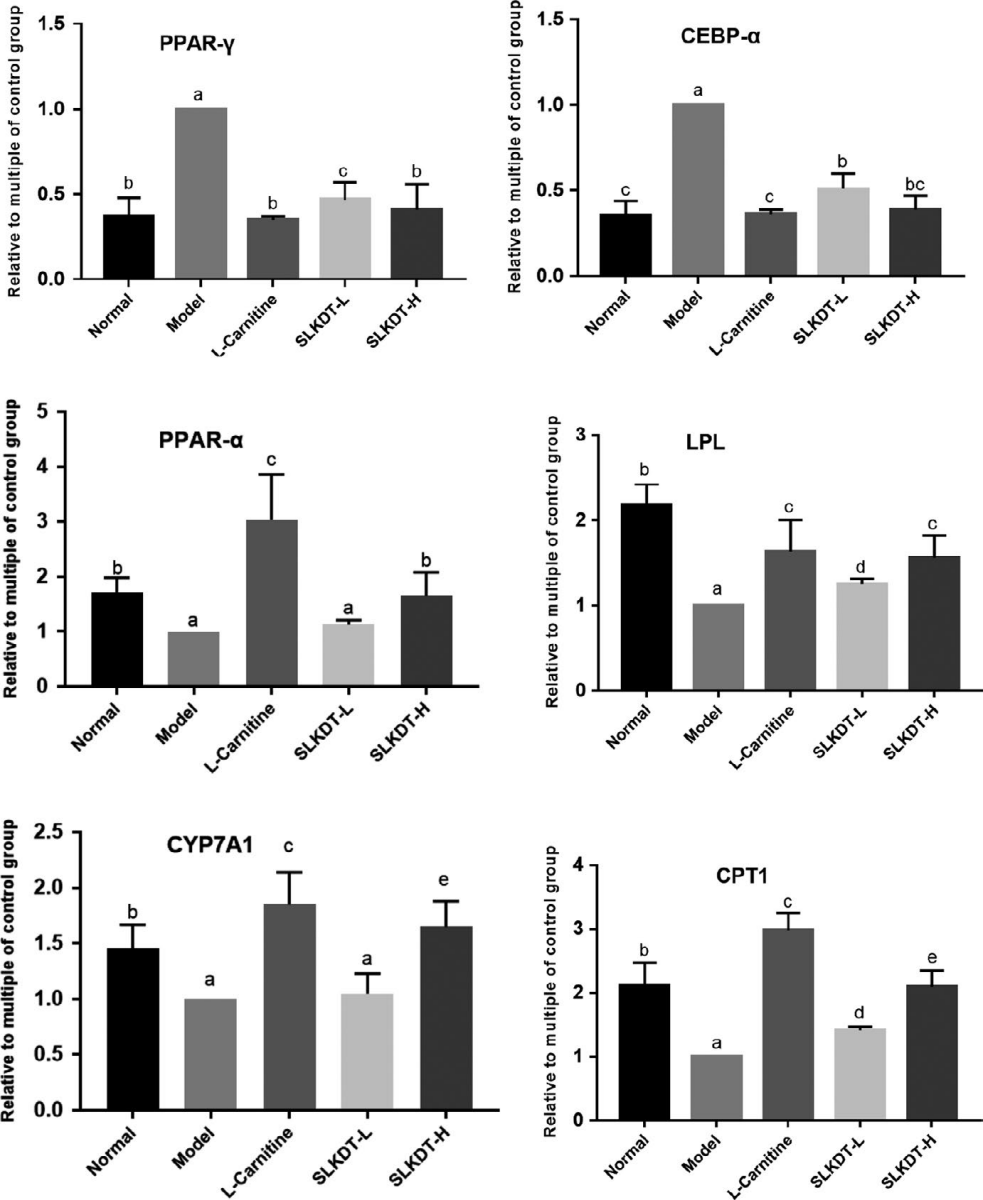
mRNA expression levels of *PPAR‐γ, C/EBP‐α, PPAR‐α, LPL, CPT1,* and *CYP7A1* in liver tissues of the different groups were investigated by RT‐qPCR. The data are shown as mean ± *SD* (*n* = 10). ^a–e^Mean values with different letters are significant difference (*p* < .05) according to analysis of variance. C/EBP‐α, CCAAT/enhances binding protein alpha; CPT1, carnitine palmitoyltransferase 1; CYP7A1, cholesterol 7 alpha hydroxylase; LPL, lipoprotein lipase; PPAR‐α, peroxisome proliferator‐activated receptor alpha; PPAR‐γ, peroxisome proliferator‐activated receptor gamma; SLKDT, small‐leaved Kuding tea

To explore the molecular mechanism of SLKDT in preventing obesity and regulating lipid metabolism in mice, we investigated the expressions of lipid metabolism‐related genes. Figure 4 shows that the high‐fat diet enhanced the mRNA expression levels of *PPAR‐γ* and *C/EBP‐α* and suppressed the mRNA expression levels of *PPAR‐α*, lipoprotein lipase (*LPL*), *CPT1*, and *CYP7A1* in the model group (*p* < .05). SLKDT‐H and L‐carnitine downregulated the *PPAR‐γ* and *C/EBP‐α* mRNA expressions and upregulated the *PPAR‐α*, *LPL*, *CPT1*, and *CYP7A1* mRNA expressions (*p* < .05). L‐carnitine had a significant effect, and SLKDT‐H was more effective than was SLKDT‐L.

## 4 DISCUSSION

The main blood lipid components are TG and cholesterol. Triglycerides are mainly involved in energy metabolism, and cholesterol is mainly used to synthesize cell plasma membranes, steroid hormones, and bile acids. Long‐term high‐fat diets cause obesity (Hedegaard, 2016), and obesity is accompanied by dyslipidemia, which involves hypercholesterolemia, hypertriglyceridemia, and reduced HDL (Kotsis, Antza, Doundoulakis, & Stabouli, 2017). Dyslipidemia is closely related to the occurrence of diseases such as atherosclerosis and hypertension (Tonstad & Després, 2011). Many recent studies have shown that drinking tea regulates lipid metabolism, reduces TGs, TC, and LDL‐C in the blood, and reduces lipid accumulation in other organs and tissues such as the liver and kidneys. Thus, tea can help lower the risks of obesity and hyperlipidemia (Chantre & Lairon, 2002; Nagao, Hase, & Tokimitsu, 2007; Nagao et al., 2015; Nishiumi et al., 2010). The anti‐obesity mechanism of tea is to inhibit the expression of adipogenesis‐related genes (Yang, Yin, Li, & Chen, 2014) and fat‐synthesis‐related enzymes (Li, Liu, et al., 2013; Sung et al., 2010; Zhang, Xiao, Wang, Wu, & Tian, 2006), promote fat decomposition (Venables, Hulston, Cox, & Jeukendrup, 2008), and reduce the decomposition and absorption of fat in food (Unno et al., 2009). Polyphenols are the key anti‐hyperlipidemic and anti‐obesity ingredients in tea (Brown et al., 2011; Uchiyama, Taniguchi, Saka, Yoshida, & Yajima, 2011). The principal polyphenols in SLKDT are chlorogenic acid and its derivatives (Wang et al., 2013). HPLC analysis indicated that the main polyphenols from SLKDT were isochlorogenic acid A, neochlorogenic acid, cryptochlorogenic acid, isochlorogenic acid B, chlorogenic acid, and isochlorogenic acid C, which matched the findings of a previous report (Wang et al., 2013). SLKDT controlled body weight gain and fat accumulation, significantly reduced the TC, TG, and LDL‐C levels, and increased the HDL‐C level in the serum and liver tissues of mice with lipid metabolism disorders. The phenolic acids may have played important roles in preventing obesity and regulating lipid metabolic disorders.

High‐fat diets cause excessive fat accumulation in hepatocytes, which causes liver steatosis that develops into non‐alcoholic fatty liver (Sookoian & Pirola, 2008). Obesity is the main cause of non‐alcoholic fatty liver. When hepatocytes are damaged, the ALT, AST, and AKP levels increase significantly, reflecting the degree of liver damage Wong, Bach, Sun, Hmama, & Av‐Gay, 2011). SLKDT reduced the liver weight and inhibited liver lipid accumulation to the same extent. Liver function test results showed that SLKDT significantly reduced the serum ALT, AST, and AKP levels to protect against high‐fat‐diet‐induced liver damage.

Studies have shown that obesity induces chronic low‐level inflammation in the body, which differs from traditional inflammation that is characterized by redness, swelling, heat, and pain (Hotamisligil, 2006; Medzhitov, 2008). The long‐term low‐level inflammatory response is closely related to various metabolic syndromes, which can induce a series of related chronic diseases such as cardiovascular disease, type 2 diabetes, and malignant tumors (Gregor & Hotamisligil, 2011; Kolb, Sutterwala, & Zhang, 2016). Adipose tissue is the earliest proven connection inflammation with obesity. In addition to storing energy, adipose tissue is an endocrine organ that secretes many hormones and cytokines such as leptin, adiponectin, resistin, and inflammatory factors (Hotamisligil, 2017; Hummasti & Hotamisligil, 2010). Hypertrophic adipose tissue increases the release of free fatty acids and inflammatory mediators (e.g., TNF‐α, IL‐6, IL‐1, IFN‐γ, and monocyte chemotactic protein‐1 [MCP‐1]), causing the tissue to become inflamed (Hotamisligil, 2017; Hummasti & Hotamisligil, 2010; Oh & Olefsky, 2012). Secreted cytokines can also activate immune cells and adjacent cells via the c‐Jun N‐terminal kinase and IκB kinase β/nuclear factor‐κB signaling pathways to increase synthesis and secretion of chemokines, such as MCP‐1, leading to proinflammatory macrophage infiltration (Oh & Olefsky, 2012). Compared with the normal group, the serum levels of proinflammatory factors TNF‐α, IFN‐γ, IL‐6, and IL‐1β in the model group were significantly increased, while the levels of anti‐inflammatory factors IL‐4 and IL‐10 were decreased, indicating that the model group mice were in an inflammatory state. SLKDT intervention significantly inhibited obesity‐induced inflammation by decreasing the proinflammatory factors IL‐6, TNF‐α, IFN‐γ, and IL‐1β and increasing the anti‐inflammatory cytokines IL‐4 and IL‐10.

CCAAT/enhancer‐binding protein‐alpha (C/EBP‐α) (Kaestner, Christy, & Lane, 1990; Liu et al., 2017) and peroxisome proliferator‐activated receptor‐γ (PPAR‐γ) (Hu, Tontonoz, & Spiegelman, 1995; Kang et al., 2018; Mueller et al., 2002) are the main transcription factors that induce mesenchymal stem cells to differentiate into adipocytes. C/EBP‐α and PPAR‐γ induce each other's expression and cooperate in activating targeted genes (Madsen, Siersbæk, Boergesen, Nielsen, & Mandrup, 2014; Rosen et al., 2002). PPARs regulate the expression of downstream genes that participate in lipid synthesis, storage, and catabolism by combining with PPAR response factors (Gross, Pawlak, Lefebvre, & Staels, 2017; Hu et al., 1995; Kang et al., 2018; Mueller et al., 2002). PPAR‐γ activation promotes differentiation and proliferation of adipocyte, stimulates fat synthesis and storage (Xu et al., 2018). Conversely, PPAR‐α positively regulates lipid metabolism by accelerating β‐oxidation of fatty acids, ketogenesis, and fatty acid intake to reduce fat accumulation (Ma et al., 2011; Ge et al., 2016).

Carnitine palmitoyltransferase I (CPT‐1) transports long‐chain acyl‐CoA to the mitochondrial inner membrane and is a key rate‐limiting enzyme in β‐oxidation of fatty acids (Huang et al., 2010). Upregulation of CPT‐1 promotes β‐oxidation of fatty acids to reduce fat accumulation. As a rate‐limiting enzyme involved in hydrolyzing TG, LPL catalyzes TG decomposition from chylomicron and very LDL into fatty acids and promotes increased HDL levels to regulate lipid metabolism (Graham et al., 2017; Tian et al., 2014). The genes that encode these two enzymes are the target genes of the PPARs signaling pathway (Ashish, Rader, & Millar, 2010; Niu, Yuan, & Fu, 2010; Yang et al., 2018), which can affect lipid metabolism and deposition. Cholesterol 7 alpha hydroxylase (CYP7A1) regulates the conversion of cholesterol into bile acid in the liver and is the key enzyme in maintaining cholesterol and bile acid homeostasis (Liu, Pathak, Boehmie, & Chiang, 2016). SLKDT downregulated *PPAR‐γ* and *C/EBP‐α* mRNA expressions and upregulated *PPAR‐α*, *CPT‐1*, *LPL*, and *CYP7A1* mRNA expressions to reduce adipocyte differentiation and fat accumulation, accelerate fat oxidation, and improve dyslipidemia, then inhibit the immune response and alleviate liver injury.

In conclusion, we systematically studied the effects of SLKDT on high‐fat‐diet‐induced obese mice, including the ability of SLKDT to prevent obesity, modulate dyslipidemia and inflammation accompanied by obesity, and prevent liver damage due to lipid metabolic disorders. SLKDT extracts upregulated mRNA expressions of *PPAR‐α*, *LPL*, *CPT1*, and *CYP7A1* and downregulated mRNA expressions of *PPAR‐γ* and *C/EBP‐α*, which reduced adipocyte differentiation and fat accumulation, promoted fat oxidation, and improved dyslipidemia. SLKDT shows great potential in preventing obesity and regulating obesity‐related syndrome, which is conceivable to be further developed into anti‐obesity product.

## CONFLICT OF INTEREST

The authors of this manuscript state that they do not have conflict of interest to declare.

## ETHICAL APPROVAL

This study was approved by the Ethics Committee of Chongqing Collaborative Innovation Center for Functional Food (201907031B), Chongqing, China.
